# From health literacy to capacity preservation: advancing the next step in midlife frailty prevention

**DOI:** 10.1016/j.tjfa.2026.100182

**Published:** 2026-06-29

**Authors:** Sitti Johri Nasela, Zulfikar Peluw, Muhammad Taufan Umasugi

**Affiliations:** a Department of Community Nursing, Poltekkes Kemenkes Maluku, Jln, Laksdya Leo Wattimena KM.16, Waiheru 97233 Ambon, Indonesia; b Department of Nursing Management, Poltekkes Kemenkes Maluku, Jln, Laksdya Leo Wattimena KM.16, Waiheru 97233 Ambon, Indonesia; c Department of Basic Nursing & Nursing Education, STIKes Maluku Husada, Jl. Kebun Cengkeh, Batu Merah, Kec. Sirimau Kota Ambon 97128. Indonesia; d Doctoral Program, Fakultas Kedokteran, Kesehatan Masyarakat & Keperawatan. Universitas Gadjah Mada, Jalan Farmako, Sekip Utara, Bulaksumur, Depok, Yogyakarta 55281, Indonesia

Dear Editor,

We read with great interest the article by Yu et al. entitled “Preparation for healthy ageing: An integrated educational intervention for enhancing knowledge and self-efficacy in intrinsic capacity preservation, midlife condition management, and caregiving in midlife women” [1]. The authors should be commended for positioning midlife as a meaningful window for healthy ageing intervention, rather than treating frailty prevention as a concern confined to advanced age. Their programme is particularly valuable because it integrates intrinsic capacity (IC) preservation, self-care and caregiving competencies within a community-based educational model.

The study makes an important contribution to the frailty and ageing literature by shifting attention upstream. In many health systems, frailty is still recognised only after functional decline, falls, hospitalisation or dependency have occurred. By contrast, the intervention evaluated by Yu et al. suggests that midlife women may represent a strategic population for preventive education, especially as they often occupy a dual role: individuals preparing for their own ageing trajectory and informal caregivers supporting ageing relatives [1]. This dual-capacity perspective is timely and consistent with the broader movement from disease-centred care towards person-centred and function-oriented ageing care [2].

However, we propose that the next conceptual and methodological step is to move from educational effectiveness to capacity translation. Improvements in knowledge and self-efficacy are necessary, but they are not sufficient to establish whether an intervention preserves IC, delays frailty, or modifies clinically relevant trajectories. The authors acknowledge the absence of longer-term behavioural and clinical outcomes. We suggest that future studies should explicitly test the pathway: health literacy → self-efficacy → behaviour change → IC preservation → frailty prevention. Without this causal chain, there is a risk that educational gains may be interpreted as preventive efficacy, although the actual behavioural and physiological translation remains uncertain.

This point is especially relevant because IC is increasingly recognised as a predictor of functional decline and mortality in older adults [3]. If IC is to serve as a bridge between midlife health promotion and later-life frailty prevention, intervention studies should include longitudinal IC outcomes, frailty status, physical performance, nutritional status, healthcare utilisation and participation in daily activities. A recent mixed-methods multicomponent intervention in midlife women has shown that IC can be measured as a modifiable outcome in this population, including locomotor and vitality-related domains [4]. Such evidence suggests that educational programmes may be strengthened by embedding objective or semi-objective indicators alongside self-reported confidence.

A second issue concerns the caregiving dimension. The intervention appropriately recognises caregiving as a core midlife responsibility, yet caregiving confidence was measured only after the intervention. Future work could benefit from a dyadic or family-centred design, assessing not only the caregiver perceived competence but also whether caregiving behaviours influence the functional ability, safety, dignity and service use of care recipients. This would extend the programme from individual education to an intergenerational model of capacity preservation.

Third, implementation equity deserves closer attention. The study sample was highly educated, which may limit generalisability to women with lower literacy, fewer resources, migrant backgrounds, rural residence or limited access to structured community programmes. Since frailty is shaped not only by biology but also by social disadvantage, future interventions should test whether IC-focused education remains effective when adapted for populations with lower health literacy, financial constraints or heavier caregiving burden. The updated Medical Research Council framework for complex interventions emphasises the importance of context, implementation, mechanisms and equity when evaluating real-world interventions [5]. This lens may be particularly useful for the next phase of IC-based community programmes.

Overall, the article by Yu et al. [1] provide a promising foundation for rethinking frailty prevention before old age. Their work invites a broader research agenda: not only how to educate midlife adults, but how to convert education into sustained behaviour, measurable capacity preservation and equitable healthy ageing. In this sense, the central question is no longer simply whether women know more after an intervention, but whether such knowledge can alter the life-course trajectory through which vulnerability becomes frailty.

## Authorship / credit authorship contribution statement

Sitti Johri Nasela: Conceptualization, Methodology, Writing – original draft.

Zulfikar Peluw: Data curation, Formal analysis, Visualization, Writing – original draft.

Muhammad Taufan Umasugi: Conceptualization, Writing – original draft, Writing – review & editing, and final approval of the manuscript.

## Use of artificial intelligence

AI-assisted language tools were used solely for grammar and stylistic editing under the author’s direction. The author verified all content and accepts full responsibility for the accuracy and integrity of the manuscript. No AI system was used to generate scientific claims, analyse data, or draw conclusions.

## Declaration of the use of generative AI

During the preparation and revision of this manuscript, the author used ChatGPT (OpenAI) & Grammarly solely to assist with language editing, formatting consistency, and the preparation of journal-required declaration sections. No generative AI or AI-assisted technology was used to generate research data, analyse data, interpret findings, create figures, images, or artwork, or replace the author’s scientific judgement. The author reviewed, edited, and approved all AI-assisted output and takes full responsibility for the content of the manuscript.

## Funding

No external funding or financial support was received for the preparation of this manuscript.

## Declaration of competing interest

The authors declare that they have no known competing financial interests or personal relationships that could have appeared to influence the work reported in this paper.
